# Level of Concern, Spending, and External Support Related to COVID-19: A Comparison between Working and Non-Working Older Adults

**DOI:** 10.3390/ijerph191811375

**Published:** 2022-09-09

**Authors:** Zuojin Yu, Aurora B. Le, Alexa Doerr, Todd D. Smith

**Affiliations:** 1 Department of Health Sciences, Towson University, Towson, MD 21252, USA; 2 Department of Environmental Health Sciences, School of Public Health, University of Michigan, Ann Arbor, MI 48109, USA; 3 KBR Wyle Services, LLC, NASA Johnson Space Center, Houston, TX 77058, USA; 4 Department of Applied Health Science, School of Public Health, Indiana University—Bloomington, Bloomington, IN 47405, USA

**Keywords:** COVID-19, lifestyle, work, older adults, support, spending

## Abstract

This study compared levels of concern, spending, and use of external support by working status among older adults in the U.S. during the COVID-19 pandemic. It assessed whether work influences these variables related to wellness. Data from 2489 older adults from the 2020 U.S. Health and Retirement Study were analyzed using multiple linear and logistic regression. Older adults who worked had lower concerns about the pandemic (β = −0.28, *p* = 0.048), were less likely to increase their spending (OR = 0.74, *p* = 0.041), and were less likely to use external support (OR = 0.50, *p* < 0.001). Use of external support increased with age (OR = 1.04, *p* < 0.001) and increased spending (OR = 1.32, *p* = 0.019). Married older adults were less likely to increase spending (OR = 0.75, *p* = 0.007) and had lower concerns toward COVID-19 (β = −0.28, *p* = 0.011). Higher levels of concern were reported among women (β = 0.31, *p* = 0.005) and participants who had friends or family members diagnosed with COVID-19 (β = 0.51, *p* < 0.001). Women were more likely to use support (OR = 1.80, *p* < 0.001). Work appears to bolster older adult wellness outcomes.

## 1. Introduction

SARS-CoV-2, the virus that causes COVID-19, has had an unprecedented global impact. Since March 2020 there have been over 92 million cases of COVID-19 and over one million deaths in the United States [1]. Older adults have been disproportionately affected by severe infection, hospitalization, and death from COVID-19. Those aged 50 to 64 are four times more likely to be hospitalized and 30 times more likely to die, relative to those aged 18 to 29; those 65 to 74 are at a nine times greater risk of hospitalization and 220 times greater risk of death [2].

Despite the recognized elevated health risks, limited research has been conducted with older adults, particularly research that aims to explore older adults’ levels of concern toward COVID-19 and the impact of the pandemic on various aspects, including spending and usage of resources or support; this, in turn, impacts older adults’ attitudes regarding COVID-19 as well as their overall health and wellbeing. Previous studies have examined the support older adults received from young caregivers within the family, but they were conducted among relatively small samples [3]. Our study used nationally representative data to investigate whether the structure within the family was associated with older adults’ concern toward COVID-19, spending, and the need for external support.

Work can provide support and opportunities that promote health and well-being among older adults [4]. This was critical, especially at the start of the pandemic, when constraints on physical activity were present and social interactions were limited for older adults [5]. Despite the known benefits associated with work, recent studies have highlighted the challenges older adults encounter balancing occupational exposure risks during the pandemic. Older adults working during the COVID-19 pandemic faced elevated physical and psychological exposure, but the detrimental financial impact on non-working adults can be life-altering [6]. This was underscored when many people were required to increase spending on necessities, such as food, and use additional services, such as grocery delivery, to reduce social contact [7]. In some cases, spending significantly increased due to panic buying and hoarding behaviors [8]. Nevertheless, employment status and social and organizational support through one’s workplace can positively impact health-promoting behaviors such as seeking medical care, having a daily purpose, getting tested and vaccinated, using masks, interacting socially, and earning an income [9,10,11,12,13,14].

Furthermore, the psychological impact of COVID-19 on older adults has yet to be fully explored [15,16,17]. Literature is still emerging on the impact of social support on the mental health of older adults during the pandemic. Social distancing—a critical primary prevention method to limit the spread of COVID-19—has also resulted in increased social isolation and exacerbated depression, anxiety, suicidal ideation, and the rate of cognitive decline of older adults, especially those not working and those living in long-term residential facilities [18]. It is understood that social support is inversely related to depression, anxiety, irritability, sleep quality, and loneliness [19], illustrating the importance of examining support among older adults during the pandemic. Research exploring differences between working and non-working older adults in this context is even more limited and warrants exploration.

Although researchers are beginning to examine various outcomes of the pandemic among working and non-working older adults, there is a dearth of information related to the pandemic and factors that might influence overall health and wellbeing such as spending, use of social support, and overall concern with the pandemic. We see the population is learning to live with COVID; simultaneously, the virus has been mutating to coexist with humans. Thus, it is important for researchers to study who has more concern about the pandemic and, thereby, who may have greater difficulty in understanding the trends we are seeing. Based on the results of such studies, policy makers can properly allocate resources to assist identified vulnerable groups. A review of the literature suggests there are limited studies that touched on individuals’ perceptions regarding the COVID-19 pandemic, especially the vulnerable older adults in the United States [20,21]. This exploratory study aims to fill that gap by examining the level of concern towards COVID-19 among a sample of older adults in the United States. Our study further examined the impact of the COVID-19 pandemic on spending and usage of support in the same population. Findings may illustrate the benefits of work among this population and provide implications for participation in work to bolster healthy decision making, and provide a source of wellness during pandemics.

## 2. Materials and Methods

### 2.1. Data and Study Sample

We analyzed data from a nationally representative dataset collected through the 2020 U.S. Health and Retirement Study (HRS) COVID-19 Project [22]. The HRS is sponsored by the National Institute on Aging (grant number NIA U01AG009740) through the University of Michigan. Data included measures related to demographic information, physical health status, employment status, and family structure, as well as attitudes, behaviors, and support related to the COVID-19 pandemic. The 2020 HRS COVID-19 Project dataset included 3266 respondents. Interviews were conducted between June 2020 and November 2020. In our study, we only included respondents who were 60 years of age or older (*n* = 2495), given the high vulnerability to severe infections and mortalities among this age group [23,24].

### 2.2. Measures

Working status was captured through the survey question asking about the respondent’s current employment situation. Respondents who chose “working now” were considered as “working”, and all other responses (temporarily laid off, unemployed and looking for work, disabled and unable to work, retired, homemaker) were classified as “non-working”. Respondents were asked to rate their levels of concern about the COVID-19 pandemic on a scale from 1 to 10, with 1 being the least concerned and 10 as the most concerned. Respondents were asked how their spending changed (went up, went down, or about the same) since the beginning of the pandemic. Dummy variables were created to distinguish those who increased their spending since the pandemic. A binary survey question, “Because of the coronavirus pandemic, did anyone living outside your household, such as a parent, adult child, other relatives, or friends, help you [and your spouse/partner] with shopping for groceries, errands, rides, or chores?”, identified those who had used external support during the COVID-19 pandemic. Health status was self-rated by a multiple-choice question, “Would you say your health is excellent, very good, good, fair, or poor?”. Response options were Excellent (1), Very good (2), Good (3), Fair (4), and Poor (5). This variable was reverse coded so higher scores represented better health status.

### 2.3. Data Analysis

Stratified by employment status, means and standard deviations were calculated for continuous variables, and frequencies and percentages were calculated for categorical variables. Multiple linear regression examined the association between the level of concern about COVID-19 with working status, increased spending, and other potential covariates, including age, gender, marital status, number of children, health status, and whether the individual was living in a nursing home. Multiple logistic regression examined whether and how increased spending was associated with working status, use of external support due to COVID-19, and other covariates including age, gender, number of children, and current marital status. Another multiple logistic regression examined whether and how external support usage was associated with age, gender, number of children, marital status, living in a nursing home, and health status. Propensity score matching further examined the effect of working status on three dependent variables (COVID concern, increased spending, and usage of external support) by minimizing selection bias from the family structure (e.g., marital status, number of children), age, and education. A pairwise deletion was used in the regression analysis for cases with missing data. All analyses were conducted with Stata v14 (StataCorp LLC, College Station, TX, USA).

## 3. Results

The final study sample included 2495 older adults who were 60 years of age or older, among which 523 (20.96%) were working and 1972 (79.04%) were not working at the time the data were collected. The average age of all participants was 72.20 years, which was higher than the average age of working participants (65.82). More than half (58.64%) of the participants were women and 53.23% of the participants were married. A total of 49 (1.96%) participants were living in a nursing home, and none of them were working at the time of the interview. Self-reported health status was higher among working older adults than non-working older adults, with a mean score of 3.45 versus 2.97. The mean level of concern toward COVID-19 was 7.81/10 among all study participants. There were 466 out of 2495 (18.7%) participants. who experienced increased spending. Among 2491 participants who reported usage of external support due to COVID-19, 601 (24.13%) answered Yes (Table 1).

Analyses showed working older adults expressed less concern toward COVID-19 than non-working older adults (β = −0.28, *p* = 0.048), after adjusting for differences in age, gender, marital status, and health status. Older adults who experienced increased spending during the COVID-19 pandemic expressed higher levels of concern (β = 0.46, *p* = 0.001). Decreased concern towards COVID-19 was seen among participants who were married (β = −0.28, *p* = 0.011), who were living in nursing homes (β = −3.18, *p* < 0.001), and who had a higher self-reported health status (β = −0.010, *p* = 0.053). Higher levels of concern for COVID-19 were reported among women (β = 0.31, *p* = 0.005) and participants who had friends or family members diagnosed with COVID-19 (β = 0.51, *p* < 0.001) (Table 2).

Working older adults were less likely to experience increased spending (OR = 0.74, *p* = 0.041). Participants who used external support were more likely to report increased spending (OR = 1.32, *p* = 0.019). The likelihood of increased spending was positively associated with number of children (OR = 1.05, *p* = 0.041). Married older adults were less likely to experience increased spending during COVID-19 (OR = 0.75, *p* = 0.007) (Table 3).

Working older adults were less likely to use support from outside of the household (OR = 0.50, *p* < 0.001). Propensity score matching confirmed a significant effect of working (β = −0.08, *p* = 0.001) on reducing the likelihood of using external support, when controlling for selection bias for age, gender, marital status, number of children, and health status. The likelihood of using external support increased with age (OR = 1.04, *p* < 0.001). Participants who rated themselves healthier were less likely to use external support (OR = 0.68, *p* < 0.001). Compared with men, women were significantly more likely to use support (OR = 1.80, *p* < 0.001), whereas nursing home residents (OR = 0.32, *p* = 0.002) and married participants (OR = 0.81, *p* = 0.038) were less likely to use external support (Table 4).

## 4. Discussion

In this study, we examined the level of concern toward the COVID-19 pandemic, increased spending during the pandemic, and the use of external support during the pandemic among a sample of older adults in the United States. We also compared differences between working older adults and non-working older adults among these outcomes. Results show higher levels of concern towards COVID-19 were associated with increased spending, which was, in turn, associated with external support usage. Relative to their non-working counterparts, working older adults had lower levels of concern toward COVID-19, a lower prevalence of increased spending, and a reduced likelihood of using external support.

Some non-working older adults lost their jobs due to the COVID-19 pandemic. Loss of employment was found to be positively associated with higher levels of concern about the COVID-19 pandemic [25]. Concerns about the pandemic may differ across occupations with differing levels of health and safety exposures. For example, a study of nurses highlighted insecurity and concern about pandemic-associated and work-related risks to themselves and their families [26]. Concerns about the pandemic also differed by gender. Findings from the present study demonstrate that women were more concerned about the pandemic than men, which is consistent with findings in the United Kingdom, Australia, Austria, France, Germany, Italy, New Zealand, and the United States [27,28]. Literature shows women are more concerned about the pandemic [27], which is consistent with women being more risk-averse [29]. We determined age is not significantly associated with a level of concern. This contradicts existing evidence that older citizens have higher risks of mortality and more severe complications if infected with COVID-19 [30,31]. Given this, one would expect an elevated level of concern among people in older age groups. We further broke down the study sample by age group and found that the 65–75 years age group had higher concern than the below-65-years age group and above-75-years age group. Additionally, concerns about the pandemic differed among individuals with varying numbers of family members. Loneliness has been found to be highly associated with fear about the pandemic [32], which explains our findings that lower levels of concern were observed among married participants, participants who have more children, and participants who were working, pointing to the importance of family and social support for older adults in the United States.

Non-working older adults, relative to working older adults, reported increased spending which may be related to their elevated level of concern about the COVID-19 pandemic. There may be factors contributing to increased spending for non-working older adults that were not captured in this study, including but not limited to costs associated with extra precautions for those who had pre-existing conditions as well as costs and surcharges associated with services. For example, throughout the pandemic, there has been a decline in usage and supply of public transportation [33]. It is possible that more non-working individuals, relative to their working counterparts, are not able to drive and, thus, rely on services such as ride share and other forms of transportation. Several studies have found that older adults’ mobility patterns changed after retirement, with fewer being likely to drive [34,35,36]. Individuals who were unable to drive and unable to get to a grocery store easily may also have had to utilize newer types of support resources (e.g., grocery delivery, food delivery, ride shares to doctors’ visits if others in their social circle are practicing physical distancing) more frequently than they did before the pandemic. Another potential reason for increased spending among non-working older adults is that many older adults experienced a “forced” adoption of e-commerce companies and social networking [37]. With increased time at home and limited options for shopping and socializing, older adults turned to the internet to meet these needs. Studies have found online shopping among older adults did indeed increase during COVID-19 [37,38]. Lastly, it should be noted that the present study found that increased spending was associated with more external support during the pandemic.

Our findings demonstrated that older participants were more likely to use external support and those who self-reported being healthier were less likely to use support. This aligns with the literature as those who are older may experience greater levels of physical and cognitive decline that require them to seek assistance to perform routine, everyday activities [39,40]. Furthermore, compared to men, women were more likely to receive support. This is concordant with the gender dynamics in the U.S. and may be attributed to the reality that men perform fewer chores than women, in general, and therefore self-report less support or assistance with chores or that women were more likely to vocalize needing assistance [41,42]. Additionally, older adults who were married or residing in a nursing home were found to use less external support. Married older adults can get assistance from their spouse/partner to help with activities and chores rather than seeking external support. While nursing homes of different types (e.g., assisted living facilities, skilled nursing facilities, independent living apartments) did suffer from staff shortages, like all industries, during the COVID-19 pandemic, there was still staff available to assist older adults to varying degrees with day-to-day functions [43]; older adults outside of these types of facilities may not have had comparable assistance [43].

As previously discussed, older adults who were currently employed at the time of the interview were less likely to use external support due to COVID-19. This may be associated with our finding that working older adults reported lower concerns about the COVID-19 pandemic relative to those who were not working during the time of the interview. Though information about why working older adults were less likely to receive support was not directly collected in the study, it makes sense that if working older adults were less concerned about the pandemic, they would be less likely to ask for help or appear to need help from friends or family members. Besides, older adults who were working are likely younger and in better health than their non-working counterparts, who may be unable to work due to pre-existing conditions, disorders, or ailments that may also make them more vulnerable to COVID-19 complications. Research has found that the higher the age of an individual, the higher the risk of being infected with COVID-19 and of dying from COVID-19, and this effect is amplified with previously diagnosed diseases [44]. Overall, older adults who were still able to work at the time of the study may have been more capable of taking care of themselves and in better health than those who were not working. Corresponding resources should be allocated to support subgroups of older adults identified in this study with a higher likelihood of using external support, given that the social support they received due to COVID-19 might not be sustainable as the coronavirus pandemic still has no end in sight.

The battle against COVID-19 has taken much longer than the public initially expected. Many countries are transitioning toward living with COVID-19 by containing and reducing instances of COVID-19, either with reduced incidence rates or diminished complications. Older adults are a widely recognized vulnerable group to the SARS-CoV-2 virus. To better assist this vulnerable group during this transition, it is important to investigate their level of concern about the pandemic as well as potential factors contributing to concerns including working status, spending, and using social support. This study concluded that increased spending was a significant predictor of elevated concern toward COVID-19. In this study, we further examined potential contributing factors of increased spending and concluded external support usage to be a significant contributor of increased spending. Our findings also demonstrated that working older adults compared to non-working older adults had lower levels of concern towards COVID-19.

This study is not without its limitations. The cross-sectional design of this study restricted our ability to conclude causal relationships. Given the data were secondary, the research was limited to only using the existing variables in the dataset. Statistical models may have omitted unmeasured factors such as race and working place (remote or on-site) in the secondary data obtained from the Health and Retirement Study. However, it is unlikely that these unmeasured factors would substantially change the major findings of our study. The features of the secondary data also limited the possibility of including control groups. We used propensity score matching to introduce matched comparisons between working and non-working groups. Future studies could further investigate the reasons for differences identified between working and non-working groups. We initially attempted to investigate what happened to those participants who lost their jobs due to COVID-19, but we were not able to obtain a valid result due to excessive missing values in the related variable. Due to the rapid-changing nature of the COVID-19 pandemic, some factors such as vaccination rate have changed significantly since the data were collected and initially published in November 2020 and updated in February 2021. Our study utilized the most up-to-date version of a reputable national survey, but it was at the time when COVID-19 vaccines had only just begun to roll out in the U.S. and vaccination information among survey participants was not widely available. Given the constant changing nature of the pandemic, future studies should further investigate how vaccination status impacts older adults’ attitudes about the COVID-19 pandemic, spending, and external support when more recent data becomes available.

## 5. Conclusions

Older adults (i.e., over 60 years old) are a highly vulnerable group to SARS-CoV-2. To effectively and efficiently allocate resources to support this group, it is important to investigate what factors contribute to their levels of concern toward the pandemic, and the financial impact and support older adults experienced due to COVID-19. By analyzing secondary data from HRS, our study concluded that increased spending was positively associated with the level of concern towards the pandemic. We further concluded that the use of external support was positively associated with increased spending. Our study also found working older adults expressed lower concerns about COVID-19 than their non-working counterparts. Compared to non-working older adults, working older adults were less likely to experience increased spending during the COVID-19 pandemic and less likely to use external support due to COVID-19. Resources should be allocated to support older adults, such as those unable to work identified in this study, to ease their concern toward the pandemic and attend to their needs for financial and social support during the ongoing coronavirus pandemic.

## Figures and Tables

**Table 1:** Descriptive Statistics of Study Sample by Working Status.

	Mean (SD) or Frequency (%)			
	Working (*n* = 523)	Non-Working (*n* = 1972)	All Sample (*n* = 2495)	*p*-Value *
**Gender**				*p* < 0.001
Male	256 (48.95%)	776 (39.35%)	1032 (41.36%)	
Female	267 (51.05%)	1196 (60.65%)	1463 (58.64%)	
**Married**				*p* < 0.001
No	207 (39.58%)	960 (48.68%)	1167 (46.77%)	
Yes	316 (60.42%)	1012 (51.32%)	1328 (53.23%)	
**Age**	65.82 (5.73)	73.90 (8.96)	72.20 (9.01)	*p* < 0.001
**Number of Children**	2.91 (1.87)	3.36 (2.20)	3.27 (2.14)	*p* < 0.001
**Health Status**	3.45 (0.91)	2.97 (1.02)	3.07 (1.01)	*p* < 0.001
**COVID-19 Concern**	7.61 (0.12)	7.86 (0.06)	7.81 (0.05)	*p* = 0.028
**Spending since COVID-19**				*p* = 0.002
Went up	80 (15.33%)	386 (19.61%)	466 (18.17%)	
Went down	134 (25.67%)	379 (19.26%)	513 (20.60%)	
About the same	308 (59.00%)	1203 (61.13%)	1511 (60.68%)	
**Used External Support**				*p* < 0.001
No	470 (90.04%)	1420 (72.12%)	1890 (75.87%)	
Yes	52 (9.96%)	549 (27.88%)	601 (24.13%)	

* Based on binary chi-square or *t*-tests for differences between working and non-working groups.

**Table 2:** Multiple Regression Predicting Participants’ Level of Concern towards COVID-19.

	Coefficient	95% CI	*p*-Value
**Working**			
No	base		
Yes	−0.28 *	(−0.56, 0.00)	0.048
**Age**	0.01	(−0.01, 0.02)	0.260
**Gender**			
Men	base		
Women	0.31 **	(0.09, 0.53)	0.005
**Living in Nursing Home**			
No	base		
Yes	−3.18 ***	(−3.96, −2.41)	<0.001
**Number of Children**	−0.07 **	(−0.12, −0.02)	0.008
**Married**			
No	base		
Yes	−0.28 *	(−0.50, −0.06)	0.011
**Health Status**	−0.10	(−0.21, 0.00)	0.053
**Family or Friends Diagnosed with COVID-19**			
No	base		
Yes	0.51 ***	(0.29, 0.73)	<0.001
**Spending Increased**			
No	base		
Yes	0.46 **	(0.19, 0.73)	0.001
**Income Reduced**			
No	base		
Yes	0.22	(−0.08, 0.53)	0.156

* *p* < 0.05, ** *p* < 0.01, *** *p* < 0.001.

**Table 3:** Logistic Regression Predicting Spending Increase during COVID-19.

	Odds Ratio	95% CI	*p*-Value
**Working**			
No	base		
Yes	0.74 *	(0.56, 0.99)	0.041
**Age**	0.99	(0.98, 1.00)	0.060
**Gender**			
Men	1.00		
Women	1.11	(0.89, 1.38)	0.353
**Number of Children**	1.05 *	(1.00, 1.10)	0.041
**Married**			
No	1.00		
Yes	0.75 **	(0.60, 0.92)	0.007
**External Support Usage**			
No	base		
Yes	1.32 *	(1.05, 1.67)	0.019

* *p* < 0.05, ** *p* < 0.01.

**Table 4:** Logistic Regression Predicting External Support Usage due to COVID-19.

	Odds Ratio	95% CI	*p*-Value
**Working**			
No	base		
Yes	0.50 ***	(0.36, 0.69)	<0.001
**Age**	1.04 ***	(1.03, 1.06)	<0.001
**Gender**			
Men	base		
Women	1.80 ***	(1.46, 2.22)	<0.001
**Living in Nursing Home**			
No	base		
Yes	0.32 **	(0.15, 0.66)	0.002
**Number of Children**	1.02	(0.97, 1.07)	0.418
**Married**			
No	base		
Yes	0.81 *	(0.66, 0.99)	0.038
**Self-rated Health Status**	0.68 ***	(0.62, 0.75)	<0.001

Note. * *p* < 0.05, ** *p* < 0.01, *** *p* < 0.001.

## Data Availability

Data used in this study can be publicly downloaded at Health and Retirement Study: https://hrsdata.isr.umich.edu/data-products (accessed on 14 July 2022).
